# The Liability of Hospital Trustees

**Published:** 1899-01-21

**Authors:** 


### The Institutional Workshop.

### THE LIABILITY OF HOSPITAL TRUSTEES.

In reference to the interesting and important question raised by "A Barrister" in our issue of December 31st, under the above heading, we have received a letter from the Hon. Sydney Holland, in which he says: "Your barrister is wrong in his law. Hospital committees are not liable for the negligence of anyone in a hospital holding any professional qualification. I happen lately to have taken legal advice on the point from Mr. Houghton and Sir R. Reid. If I can lay my hands on the opinion I will send it you." He then goes on to mention an accident that had happened at the London, and adds: "I thought we must be liable. Houghton, a very good opinion, advised not, but I was not satisfied, and so took Sir R. Reid's, and he agreed with Houghton. I showed both these to —— (the solicitor in the case), who discontinued the action." The following are the documents which we received:

"Re Liability of the Hospital.

"A patient was admitted to the London Hospital suffering from a slight injury. The patient was prepared for a surgical operation, and an anæsthetic was given to him. The ordinary ward bottle which usually contains ether pure was used, but by a mistake in the dispensary chloroform had been mixed with the ether. Given as ether, this anæsthetic proved fatal. An inquest has been held on the case, and the verdict of the jury was that 'death was due to the administration of an anæsthetic, chloroform and ether, under a mistake for ether—death by misadventure.' The widow, who has three children under fifteen years of age, now claims compensation from the hospital.

"1. Is the hospital as a corporate body responsible?

"2. Is the hospital responsible for the death of a person being treated gratuitously in the wards?

"We would submit that the hospital takes all reasonable precautions in having regulation bottles for ether and chloroform; that as a hospital it can only employ skilled dispensers, and that the committee have no control over the way medicines or anæsthetics are made up. The doctor who gave the anæsthetic said at the inquest that, without being warned, he did not perceive any smell of chloroform, but that if he had thought that it might be chloroform he might by smelling have detected it.

"Mr. Boydell Houghton has given an opinion, sent herewith, that the hospital is not liable, basing his opinion, as I understand it, on the ground that the relation of master and servant does not exist between the hospital and the paid dispenser, distinguishing a dispenser from, say, a coachman, on the ground, I presume, that in the case of a coachman the master is responsible for the manner in which he does his work; whereas, provided the dispenser is qualified and care taken in his appointment, the hospital has no control over how he does his work, and that the negligence in this case was in the discharge of the technical part of his work.

"With this opinion I venture to differ, and should be obliged if Mr. Lawson Walton would see Mr. Houghton and discuss the matter and advise the hospital. I consider the distinction too thin to be of any avail. The master of a coachman may know no more about driving than the committee of the hospital do about dispensing. I have never heard it suggested that a good defence to an action for running into the plaintiff's carriage would be 'I am not liable, I have not the remotest idea how to drive. I tell my coachman to drive carefully and skilfully, but whether he is doing so or not I cannot tell.'—I am, yours faithfully,

"Sydney Holland, Chairman."

---

Opinion.

"I am of opinion that the governing body of the London Hospital is not liable for the negligence of a member of the medical or surgical staff in the exercise of his professional duties, provided reasonable care was taken before such member was appointed—*i.e.*, that the person was duly qualified and that inquiry had been made into his antecedents, or, at all events, that there was evidence before the governing body which reasonably satisfied them that there was nothing in his previous career to suggest that he was incompetent or careless. The immunity from liability on the part of the hospital in such circumstances does not rest upon the governing body being a corporation, but upon the principle that the relation of master and servant does not exist. Although the governors select and may dismiss the staff and may pay some of the members, they have no power of 'controlling the work,' *i.e.*, the members of the staff are not bound to obey an order of the governing body as to whether or when an operation shall be performed or how it shall be carried out or what medicine shall be prescribed or how the prescription shall be made up. The precise case of a hospital has not, so far as I have been able to ascertain, been decided in the English Courts, but it has been decided in favour of a hospital in America in Glavin v. Rhode Island Hospital (34 'American Reports,' 675), and in the Court of Appeal in New Zealand in 1872 (see District of Auckland Hospital v. Lovett, 10 'New Zealand Law Reports,' p. 597). The latter was a case of negligence by a paid medical superintendent, and the hospital was held not liable, and upon reasoning which is, in my opinion, unanswerable.

"I can see no evidence of negligence upon the part of the doctor, but if there was negligence the hospital is not liable, for the reasons above stated.

"Assuming, however, that the negligence was on the part of the dispenser, and that he is paid by, and as to hours of attendance and *general* conduct under the control of the governing body, the reasoning which applies to the medical and surgical staff, in my opinion, applies equally to the dispenser in the exercise of his skilled and technical duties.

"(Signed) Boydell Houghton.

"1, Temple Gardens, Temple, 18th December, 1897."

---

Opinion of Mr. Lawson Walton, Q.C., M.P.

"I agree in substance with the opinion expressed by Mr. Houghton and for the reasons given by him.

"The case involves considerations of some nicety, but the true view of the question submitted to me is that the dispenser of a hospital is in the same category as the members of the medical staff, and that in the discharge of duties connected with the compounding and dispensing of drugs there is no relationship of superior and inferior, or master and servant, which would make the maxim of respondent superior so apply as to create liability on the part of the hospital board for errors committed.

"(Signed) J. Lawson Walton.

"Temple, January 14th, 1898."

We need hardly point out how extremely important is the question raised. Nor need we hesitate to say that, looking at the matter from the point of view of the juryman rather than the barrister, Mr. Sydney Holland's simile of the coachman appeals strongly to our common sense. Assuming, however, as we are bound to do, that the above opinions are correct in law, we are by no means sure that questions of fact do not arise which might put a very different aspect on such a case as is described. It is to be noted that Mr. J. Lawson Walton says, "The true view of the question submitted to me is that the dispenser of a hospital is in the same category as the members of the medical staff." But is he? Here we come to a question of fact. The medical staff have an absolutely free hand in the exercise of their professional discretion. But with the dispenser there is no question of the exercise of discretion. He is the paid servant of the institution, paid to do certain things in a certain way; and the question is, if he fails to do them the right way who is responsible? We do not think the question is quite settled yet.

